# Comparative Evaluation of Debris Expulsion Beyond Apex During Re-root Canal Treatment by Utilizing Two Re-treatment Rotary Files and Two Reciprocating Files: An In-Vitro Study

**DOI:** 10.7759/cureus.56467

**Published:** 2024-03-19

**Authors:** Akhila Raj. R, Iswarya Raju, Joel G Varghese, Ourvind Jeet Singh Birring, P. Jeason Paul Yacob, Hitesh Chohan

**Affiliations:** 1 Conservative Dentistry and Endodontics, Amrita School of Dentistry, Amrita Viswavidyapeetham, Kochi, IND; 2 Conservative Dentistry and Endodontics, The Muslim Educational Society (MES) Dental College, Malappuram, IND; 3 Conservative Dentistry and Endodontics, Educare Institute of Dental Sciences, Malappuram, IND; 4 Conservative Dentistry and Endodontics, Bisha Dental Center, Bisha, SAU; 5 Restorative Dental Sciences, College of Dentistry, Jazan University, Jazan, SAU

**Keywords:** dentistry, endodontics, apical debris extrusion, reciprocating files, re-treatment

## Abstract

Aim: This study aimed to assess and compare debris quality expelled beyond the apex during re-treatment using two reciprocating and two re-treatment rotary files.

Materials and methods: A total of 80 healthy human mandibular premolars excised for orthodontic therapy were selected. Distilled water was used to sterilize the teeth before they were preserved. The canals were widened and coronal access was created so that the F2 ProTaper universal system could be used. For the obturation, we utilized gutta-percha cones and AH plus sealant. All samples were cone-beam computed tomography (CBCT) recorded. Teeth were separated into four groups (n=20); Group 1: ProTaper re-treatment, Group 2: Mtwo re-treatment, Group 3: WaveOne gold, and Group 4: Reciproc. The re-instrumented process included collecting the extruded debris in Eppendorf tubes and then drying it off by evaporating the distilled water. The weight of the tube with debris was subtracted from the weight of the tube without debris to get the dry debris weight. Post-retreatment CBCT was recorded for all samples. Mean apical debris extrusion values were obtained, after which necessary statistical analysis was obtained using SPSS Statistics, version 21 (IBM Corp., Armonk, NY) to obtain the results. A one-way analysis of variance (ANOVA) and the student t-test were utilized for statistical analysis to compare the different experimental groups. Post-hoc Bonferroni tests were used to compare several groups.

Result: No statistical difference (p>0.05) was identified in the filler material that was left over across all groups. The reciprocating files discharged more material compared with re-treatment file systems.

Conclusion: The Reciproc files expelled more waste than WaveOne gold, ProTaper re-treatment, and Mtwo re-treatment file systems. Reciprocating file systems showed better cleaning efficiency compared to the re-treatment file systems.

## Introduction

Endodontic re-treatment is mainly done to eliminate the microbiological content of teeth that have undergone unsatisfactory endodontic procedures. Regaining access to the tooth's apex is the primary objective of re-treatment, which is accomplished by removing the filling material [1,2]. While minimizing the quantity of apical extrusion, the proper re-treatment approach should be used to thoroughly remove any previously applied filling material. During re-treatment, irritants like filling materials, bacteria, and irrigants may move into the periapical area through the foramen. This could lead to pain after surgery and an increase in reactions to foreign bodies. Immunoglobulin in the apical region interacts with the antigen in the canal when its contents are pushed there. Pain, swelling, bone loss, an overactive kinin system, and other symptoms might result from this response damaging the cell membrane of good periodontal tissue [3,4].

Hand files, reciprocating Ni-Ti files, rotating Ni-Ti files, and re-treatment files are just a few of the instruments used to clean up canals after filler material has been placed there. Re-treatment files were used to remove the root canal filling materials [5]. “The ProTaper Universal Retreatment System (PTUR) (Dentsply Maillefer, Ballaigues, Switzerland) consists of D1 (30/0.09), D2 (25/0.08), and D3 (20/0.07) files, which are designed with various tapers and tip diameters. While the Mtwo re-treatment system (Mtwo R) (VDW, Munich, Germany) has two files: R1 (25/0.05) and R2 (15/0.05)” [6]. Since the notion of reciprocating movement is so new, it has not been subjected to a thorough evaluation in terms of apical debris extrusion. Understanding how the changes affect debris extrusion requires studies of apically extruded debris (AED) employing these novel re-treatment file systems. The reciprocating action (i.e., alternating clockwise and counterclockwise) is carried out by a special motor unique to WaveOne (Dentsply/Maillefer, Ballaigues, Switzerland) single file systems, which are advised for one-time usage only [7,8]. It is offered in three sizes with a set taper: small (21 tips/0.06), primary (25/0.08), and large (40/0.08). There are three different-sized fixed-taper Reciproc files available (25/0.08 for small canals, 40/0.06 for medium, and 50/0.05 for large canals).

Considering that the AED causes postoperative inflammation and slows wound healing, evaluating the efficiency of the instrumentation method and instrument design is essential [9]. When doing rotary instrumentation or re-treatment, clinicians should think about the root's anatomy and the relevance of remaining dentin thickness (RDT). Cone beam computed tomography (CBCT) has been a godsend in this respect since it allows for the measurement of dentin thickness in root canal walls. As a result, the RDT can be evaluated with great precision thanks to its non-invasive 3D view, accuracy, and dependability [10]. 

Previous studies have emphasized the importance of endodontic re-treatment to eliminate microbiological content from teeth that have undergone unsatisfactory endodontic procedures [11-14]. During endodontic treatment, debris may be extruded into the periapical tissues in measurable amounts, and several experimental setups have been developed to measure this. In their description, Myers and Montgomery detailed the use of a rubber stopper, a glass vial, and a glass flask, all of which have now become standard equipment for most research [15]. The assembly where the tooth is inserted should be fixed to prevent movement, and the flask should be protected using a rubber dam so that the operator is not able to view the debris, emulating a clinical working environment. Using two re-treatment file systems and two reciprocating file systems, the quantity of debris extruded apically via Myers and Montgomery was measured and compared, as was the cleaning efficiency using CBCT during re-treatment. The hypothesis being tested here is that there is no substantial variation in the quantity of debris ejected across all file systems. 

The primary objective of re-treatment is to regain access to the tooth's apex by removing previously applied filling material while minimizing apical extrusion. AED during re-treatment can lead to postoperative inflammation and hinder wound healing, making it a critical consideration. To address this gap, the text describes a study that aims to measure and compare the quantity of debris extruded apically using two re-treatment file systems and two reciprocating file systems.

## Materials and methods

Eighty premolars from human mandibles with closed apices were taken out for orthodontic purposes. The sample size was calculated using G*Power 3.1.9.2 software, with an alpha error of 0.05, power of 80%, and effective size of 0.80. This research was a joint effort between the Amrita School of Dentistry's Department of Conservative Dentistry and Endodontics and the School's Department of Nanoscience. Extracted for orthodontic and periodontal reasons, 80 single-rooted mandibular premolars were chosen for the study, and their 0-10° curvature apices were verified using digital radiography (Schick Tech Inc., Long Island City, NY, USA). Each tooth was examined from both the buccal and proximal perspectives to ensure it had a single, symmetrical root canal and fully developed roots. Teeth having root caries, calcification, and open apices were eliminated from the research.

To reduce the potential for bias due to operator variation, the whole investigation was conducted by a single operator. Mechanical procedures were used to clear the root surface of any remaining soft tissue and calculi. The teeth's buccal cusp was filed down so that it would serve as a level surface. A high-velocity bur was used to create the standard coronal access cavity. The apical foramen was exposed by inserting a 10k file with a rubber stopper into the canal. The operational length was calculated by taking this value and subtracting 1 mm. All of the samples' glide paths were saved in a 20-kilobyte file. “The root canals were prepared with the ProTaper Universal system (Dentsply Maillefer, Ballaigues, Switzerland) in a crown-down manner in combination with a torque-controlled engine (X-Smart, Dentsply Maillefer, Ballaigues, Switzerland) at 250 rpm according to the manufacturer's instructions up to F2 and obturated using gutta-percha and AH plus sealer.” Zinc oxide eugenol (ZnOE) cement was used to temporarily restore the cavities. All 80 samples were CBCT recorded. Two months were spent keeping all samples at 37° with 100% humidity.

For instrumentation using various data systems, the 80 chosen teeth were split into four groups (n = 20) at random. Group 1: for the ProTaper re-treatment file system, the sequence of instruments used was D1, D2, and D3 until the working length. Group 2: for two re-treatment file systems, R15 and R25 file systems were used until the working length. Group 3: a large WaveOne gold file was employed within and out of motion per the manufacturer’s instructions. This file was cleaned after withdrawing, and before reusing, it was checked thoroughly. Up until the file reached the workable length, the process was repeated. Group 4: an R40 Reciproc file was used as per the instructions by the manufacturer. The file was taken out of the canal, cleaned, and examined before being used again. The process was continued until the file’s length became close to the workable length. Each group used 5 mL of distilled water to irrigate the canal.

**Figure 1:**
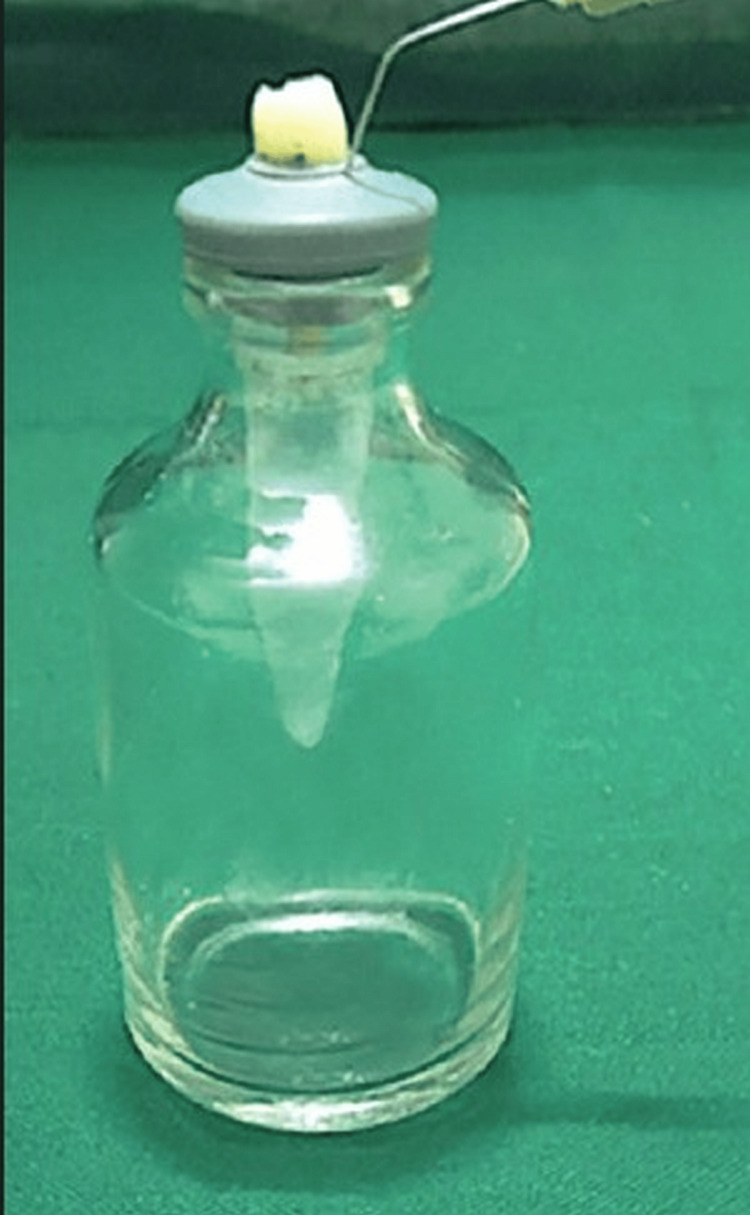
The study technique used for debris evaluation

Myers and Montgomery's analytical approach was used to assess the dumped trash at the end of the storage period [11]. For 15 days at 37° Celsius, all of the cylinders were brooded in a natural incubator to remove the surplus flooding arrangement. The logical balance (Sartorius, Germany) was used to pre-weigh the Eppendorf tubes. After opening an Eppendorf tube plug until the cementoenamel junction (CEJ) was visible 1-2 mm above the plug, a tooth was put within. Each plug was then reached with the Eppendorf tube by using the user's teeth. This strategy was written on a vial made of glass. A 27-check needle was positioned next to the plug to regulate the air pressure within and outside the cylinder (Figure 1).

**Figure 2:**
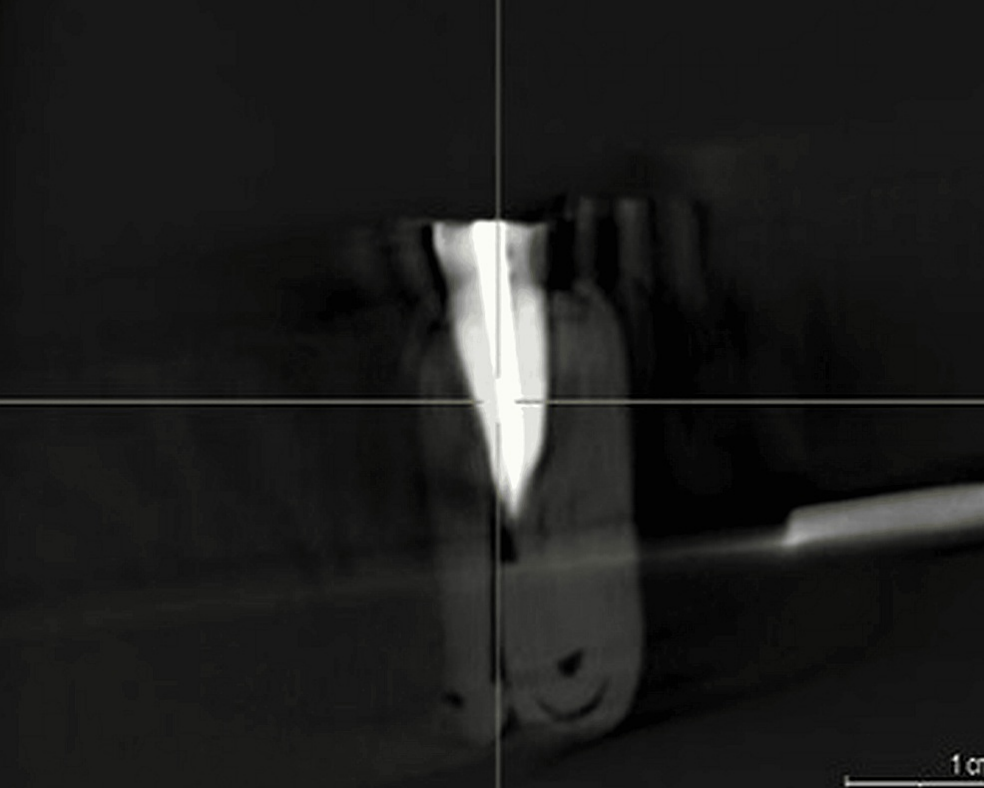
Pre-retreatment CBCT image of the premolar

**Figure 3:**
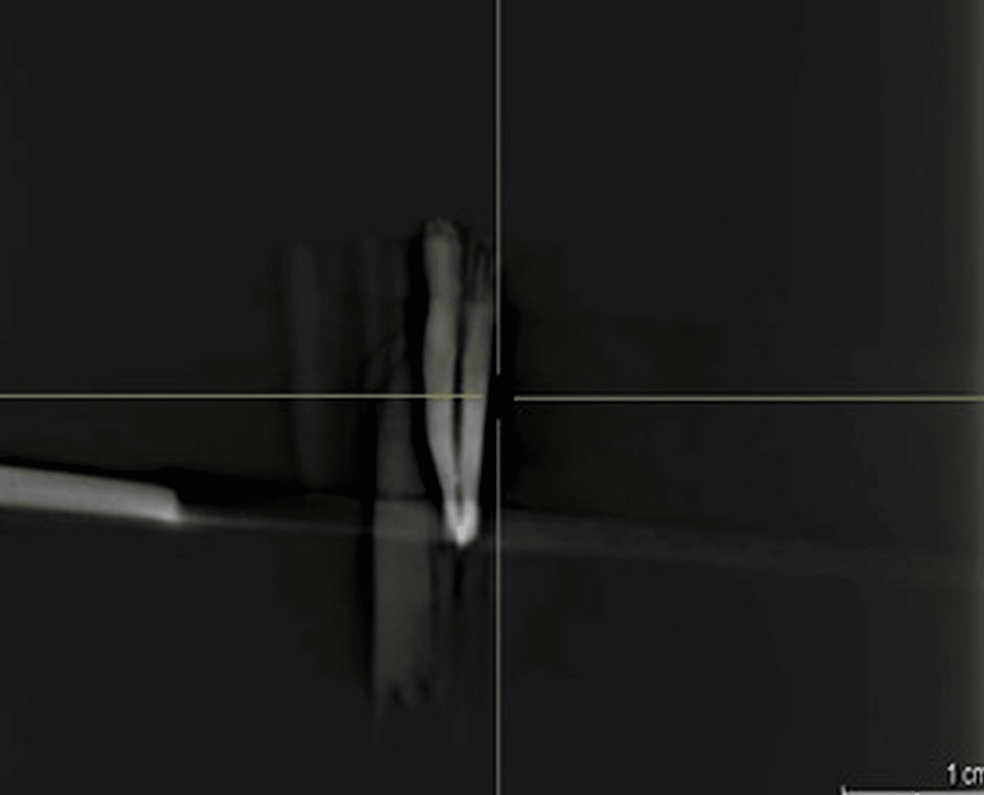
Post-retreatmnet CBCT of the premolar

Expelled waste collected in Eppendorf tubes was reprocessed after each collection, with the lyophilizer used to evaporate the purified water before determining the dry flotsam and jetsam weight. Cleared material dry weight was determined by subtracting empty cylinder weight from full cylinder weight. All events after re-treatment were CBCT recorded (Figure 2 and Figure 3).

Statistical analysis

A one-way analysis of variance (ANOVA) and the student t-test were utilized for statistical analysis to compare the different experimental groups. Post-hoc Bonferroni tests were used to compare several groups. Qualitative variables, including the experimental groups and file systems used, were essential for characterizing the different treatment conditions and procedures.

Ethical consideration

The ethical approval for the study was obtained from the Amrita School of Dentistry with institutional review board number IEC/2022/ASD/10.

## Results

Apical debris is always removed throughout re-treatment procedures. On comparing the quantity of debris extruded generated by the re-treatment file systems and the reciprocating file systems, using the student t-test, the reciprocating file systems exhibited a significant difference from the re-treatment file systems (Table 1).

**Table 1:** Comparison of apical debris extrusion (g) between Group A (re-treatment) and Group B (reciprocating) using student t-test CI, confidential interval

Group	Mean	Mean difference	95% CI			P-value	
Group A	0.000010±0.000014	2.2E-05	(0.000008-0.000035)			0.003	
								Group B	0.000032±0.000022

Maximum debris extrusion was seen using the Reciproc file system, followed by the WaveOne gold file system and the ProTaper re-treatment file system. The Mtwo re-treatment file systems showed the least amount of debris ejection (Table 2).

**Table 2:** Comparison of mean apical extrusion of debris (g) between all experimental groups using the one way ANOVA test SE, standard error; ANOVA, one-way analysis of variance

Groups		Mean difference	SE	P-value	95% CI	
					Lower bound	Upper bound
ProTaper	Mtwo	0.000014	0.000008	0.718	-0.000010	-0.000038
	WaveOne	0.000005	0.000008	1.000	-0.000029	0.000019
	Reciproc	-0.000024	0.000008	0.045	-0.000049	-0.000060
Mtwo	WaveOne	-0.000019	0.000008	0.218	-0.000043	0.000005
	Reciproc	-0.000038	0.000008	0.001	-0.000063	-0.000014
	ProTaper	-0.000013	0.000008	0.718	-0.000038	0.000010
WaveOne	Reciproc	-0.000019	0.000008	0.180	-0.000044	0.000048
	ProTaper	0.000005	0.000008	1.00	-0.000019	0.000030
	Mtwo	0.000010	0.000008	0.218	-0.000005	0.000043
Reciproc	ProTaper	0.000024	0.000008	0.045	0.000012	0.000050
	Mtwo	0.000038	0.000008	0.001	0.000014	0.000063
	WaveOne	0.000020	0.000008	0.180	-0.000004	0.000044

**Figure 4:**
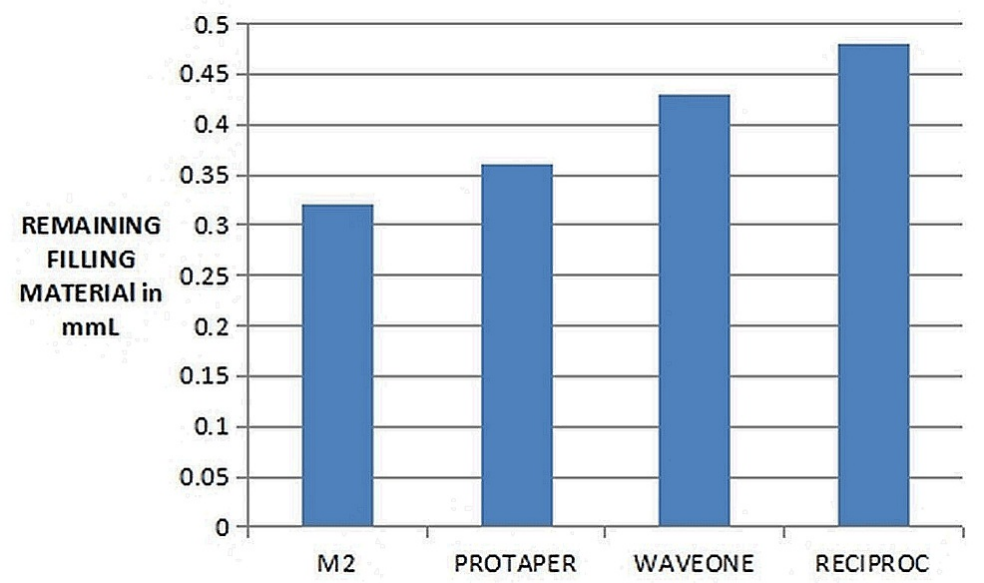
Comparison of the cleaning efficiency of the different files

There was no statistically significant difference (p > 0.05) between the volumes of remaining filling material after re-treatment between the four groups (Figure 4).

**Figure 5:**
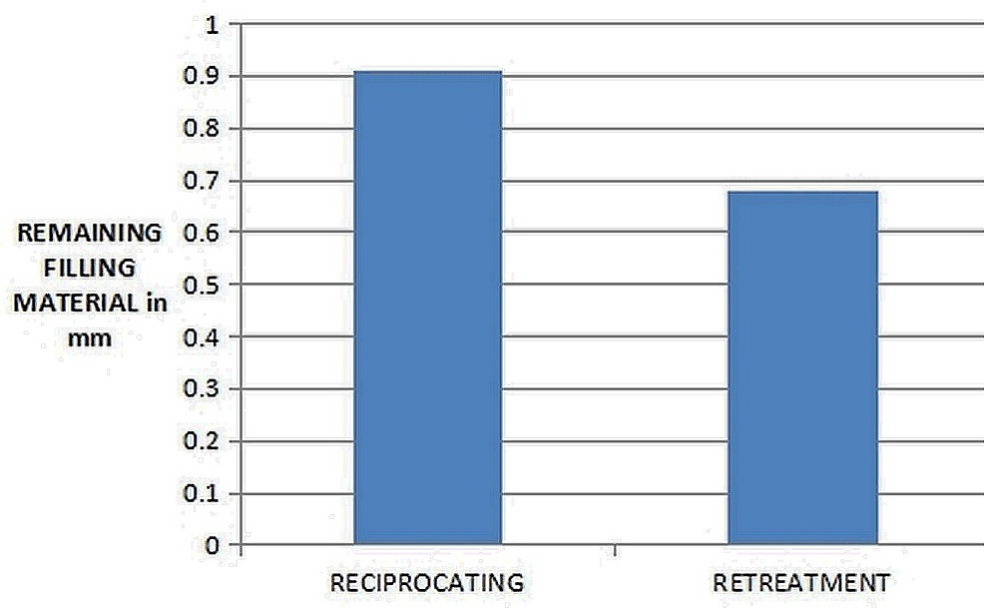
Comparison of the cleaning efficiency among the reciprocating and re-treatment groups

The least amount of remaining filling material was observed with the use of the Reciproc, followed by the WaveOne gold, the ProTaper, and finally the Mtwo re-treatment file system (Figure 5).

## Discussion

Removal of the contaminated filling material, followed by cleaning, shaping, and refilling the root canal, is the main goal of root canal retreatment. It is very uncommon for contaminated material to be extruded into the periradicular tissues during retreatment operations. During a second root canal procedure, waste material may inadvertently be pushed over the apex. Both postoperative inflammation and recovery time might be affected by the amount of material removed [12]. “Factors affecting the extrusion of debris include apical patency, dentin hardness, quantity and flow of irrigant, size of the final apical file, and instrumentation techniques” [13,14]. This research set out to do just that via a series of comparisons and assessments. This in vitro research project took mandibular first premolars into account since they are typically lost after orthodontic treatment is completed. Despite the typically straight nature of their canals, the inclusion of additional canals may affect the total amount of apical extrusion [15].

The use of solvents in retreatment has been debated, hence they were not used in this investigation. Many studies have employed solvents, and they are effective in facilitating the complete removal of residual filling material [16]. Studies have shown that the use of solvents leaves behind traces of gutta-percha and sealer on the root canal walls and within dentinal tubules [17]. The current research design does not include the use of solvents to prevent these kinds of problems. Several methods, such as a scoring system and microbalance weighing, have been used to quantify the apical extrusion of debris. The current research employed the approach developed by Myers and Montgomery because it provides more accurate measurements and is standardized and reproducible [15]. Because of its high resistance to cyclic fatigue, reciprocating files are suitable for use in the root canal. “The outcome of the investigation demonstrated that irrespective of the variation in the taper and cross-sectional design, rotary retreatment files (pro taper and Mtwo retreatment files) extruded less apical debris extrusion compared to reciprocating files (recognition and wave one gold reciprocating systems).”

Previous work by Burklein et al. indicated that rotary files were linked to reduced debris output than reciprocating files; the present studies corroborate that finding [18]. Re-treatment files have been demonstrated to be better than reciprocating files in certain research, and vice versa. Variations in canal anatomy, irrigant usage, file organization, and layout all play a role in producing varying outcomes [19]. Comparing Mtwo retreatment files to ProTaper retreatment files, we found that Mtwo files resulted in decreased extrusion of apical debris; however, this difference was not statistically significant. This result is in conjunction with the previous study by Dincer et al. [20]. The star-tightened retreatment document's triangular cross-segment provides three resources with the root channel dentinal wall, with enough room between the instrument and the walls to compel dentinal flotsam and jetsam that favors drilling of the rubbish coronally.

WaveOne gold and the corresponding documentation did not drastically differ from one another. Correspondence, however, demonstrated increased rubbish discharge at the tip of the structure. When compared to a licensed topsy-turvy cross-area, where only one front is in touch with the channel wall, the cross-segment of WaveOne gold is a parallelogram with two 85° front lines in contact with the waterway wall. Diminishing the touch area between the document and the canal wall and with a stable helical point of 24° along the dynamic length of the instrument ensures close to zero sinking. Chips have greater room to drill debris coronally, health is improved, and cutting efficiency is increased with this cross-section. [15]. The second aim of this research was to assess how well CBCT cleaned up various file systems. By scanning at each stage of the operation during re-treatment, this non-invasive technology enables 3D quantitative measurement and in-depth analysis. Both the reciprocating and re-treatment files left much less infill material on the canal walls. This result is in line with that of Zuolo et al., who showed that reciprocating files were more effective than re-treatment files for removing infill material from canal walls [21].

When compared to only using the retreatment system, Yuruker et al. discovered that using Reciproc or manual H-files additionally greatly enhanced filler material removal [22]. The ProTaper universal retreatment system's ultimate suggested instrument is its 20-size D3 file. Files of sizes 15 and 25 from Mtwo and R25 from the Reciproc, and a 25 from the original WaveOne file, were utilized. If you are looking for anything that compares to the D3 probe's end, you have found it. Mtwo has accounted for a larger proportion of remains identified apically compared to the other groups, which might be attributed to the lower tip size. One of the study's limitations was that several rotary systems were used, each having a unique combination of file numbers, taper angles, rotating speeds, and kinematics. There has been no effort to mimic the existence of the vital pulp and periapical tissues, which may operate as a natural barrier, preventing debris from extruding in an in vivo model, therefore the results may vary.

This study assessing debris quality expelled beyond the apex during re-treatment using various file systems had limitations including the use of extracted human mandibular premolars, an artificial environment with distilled water, a focus on single-rooted teeth, the use of specific obturation materials, an ex vivo approach, limited file system comparisons, a relatively small sample size, short-term assessment, and the lack of direct clinical relevance assessment, all of which may impact the generalizability and applicability of the results to real-world clinical scenarios.

## Conclusions

The current research confirms previous results that root canal instrumentation always causes debris to be extruded from the apex of the canal. Reciprocating file systems showed better cleaning efficiency compared to the re-treatment file. The smallest amount of expulsion of debris was seen with the use of the Mtwo re-treatment file system, followed by the ProTaper re-treatment file, then WaveOne gold, and finally by Reciproc.
